# Association between udder morphology and *in vitro* activity of milk leukocytes in high yielding crossbred cows

**DOI:** 10.14202/vetworld.2017.342-347

**Published:** 2017-03-22

**Authors:** Tripti Sharma, Pradip Kumar Das, Prabal Ranjan Ghosh, Dipak Banerjee, Joydip Mukherjee

**Affiliations:** Department of Veterinary Physiology, West Bengal University of Animal and Fishery Sciences, Kolkata - 700 037, West Bengal, India

**Keywords:** cows, leukocytes, milk, morphology, teat, udder

## Abstract

**Aim:**

The present investigation was conducted to study the association between udder morphology and *in vitro* activity of milk leukocytes in high yielding crossbred cows.

**Materials and Methods:**

A total of 48 healthy high yielding crossbred cows were selected for the study. The udder configuration and teat/udder morphology were recorded before milking. Milk samples (100 ml/cow) were collected aseptically. Milk somatic cell counts (SCC) and milk differential leukocyte counts were performed microscopically. Milk leukocytes (viz., neutrophils, lymphocytes, and macrophages) were isolated from milk samples by density gradient centrifugation. Phagocytic index (PI) of milk neutrophils and macrophages were evaluated by colorimetric nitro blue tetrazolium assay. Lymphocytes proliferation response was estimated by MTT assay and expressed as stimulation index.

**Results:**

There was a significant (p<0.01) positive correlation between milk SCC with mid teat diameter, teat base diameter and significant (p<0.05) negative correlation between milk SCC and the height of the teat from the ground. Milk SCC was found to be significantly (p<0.01) lower in bowl-shaped udder and higher (p<0.01) in pendulous type. Milk macrophage percentage was positively (p<0.01) correlated with udder circumference. PI of milk neutrophil was negatively (p<0.01) correlation between teat base diameter, and PI of milk macrophages was found to be positively (p<0.01) correlated with teat apex diameter. Both PI of milk neutrophils and macrophages was found to be significantly (p<0.01) lower in the animals having flat and round teat and pendulous type of udder. *In vitro* PI of milk neutrophils was found to be significantly (p<0.01) lower in flat teat. *In vitro* PI of milk macrophages was found to be significantly (p<0.01) lower in the round and flat teats compared to pointed and cylindrical teats.

**Conclusion:**

Udder risk factors such as teat shape and size, teat to floor distance, udder shape, and size may decrease the *in vitro* activity of milk leukocytes hence facilitates the incidence intramammary infections.

## Introduction

Defense of the mammary gland against mastitis-causing pathogens is mediated by several anatomical, cellular, and soluble protective factors together with other physiological and managemental factors including breed, parity, period of lactation, udder and teat morphology, age at first calving, milk leakage, udder edema, milk production, number of milk somatic cell counts (SCC), reproductive disorders, and preventive health management [1,2]. The cellular defenses include milk SCC comprising leukocytes (neutrophils, lymphocytes, and macrophages) together with epithelial cells and play a key role in the natural defense mechanism of the udder and *in vitro* activity of milk leukocytes can also be used to monitor the udder health [3-5].

Association of udder morphology with the occurrence of mastitis has already been established worldwide [6,7]. However, scanty references are available elucidating the association between the udder morphology with udder immunity, especially the activity of mammary leukocyte.

Therefore, this study is undertaken to shed some light on the association between the udder morphology and *in vitro* milk leukocyte activity.

## Materials and Methods

### Ethical approval

The experiments on animals including all procedures of this study were approved by Institutional Animal Ethics Committee (Registration number: 763/03/a/CPCSEA).

### Selection of experimental animals

A total of 48 high yielding crossbred cows of 2^nd^ to 4^th^ parity and mid lactations (150-200 days of lactation cycle) were selected from the herd of Eastern Regional Station of ICAR-National Dairy Research Institute, Kalyani, Nadia, and West Bengal, India. All the experimental animals were kept in loose housing system under the routine managemental practices followed in the institute’s herd. All the animals were screened weekly for clinical mastitis throughout the study.

### Macroscopic examination and biometry of udder

The mammary glands of all the animals were analyzed for the presence of any gross lesions. The udder configuration and teat/udder morphology were recorded following Bhutto *et al*. [6] before the morning milking. Teat and streak canal length and teat diameter from apex, mid and base of all the teats were measured using a vernier caliper during the standing condition. The teat and udder shape was documented following Shukla *et al*. [8]. The following parameters were evaluated.

Teat length (cm): Average of all four teats.
Teat apex diameter (cm): Average of all four teats.
Mid teat diameter (cm): Average of all four teats.
Teat base diameter (cm): Average of all four teats.
Teat shape: Pointed, cylindrical, round, flat.
Udder shape: Cup, round, bowl.
Udder position: Pendulous, non-pendulous.

### Milk sampling

Composite milk samples (100 ml/cow) from all four quarters were collected into sterile tubes through hand milking for consecutive 3 times with 1 month apart. Teat dipping before the collection was done with an effective teat dip solution (0.5% iodine or 4% hypochlorite) for at least 20-30 s before milking. Then, the teats were carefully scrubbed with a cotton cloth or gauze pad moistened with 70-80% ethyl alcohol.

### Estimation of milk SCC and differential leukocyte counts (DLC)

SCC and DLC of milk samples were measured microscopically [3,4].

### Isolation and culture of milk leukocytes

Isolation of milk leukocytes - viz., neutrophils, lymphocytes, and macrophages - was performed by density gradient centrifugation [3-5].

### Evaluation of *in vitro* phagocytic index (PI) of milk neutrophils and macrophages

*In vitro* PI of milk neutrophils and macrophages was evaluated by colorimetric nitro blue tetrazolium reductive assay [9].

### Evaluation of *in vitro* milk lymphocyte proliferation response

Mitogen-induced milk lymphocyte proliferation response was measured by colorimetric MTT (tetrazolium) assay [10] and expressed as stimulation index (SI).

### Statistical analysis

All analysis was performed using SYSTAT software package. The correlation between udder morphological parameters (teat length, teat diameter, height of the teat from ground) and udder immunological parameters (milk SCC, DLC and *in vitro* activity of milk leukocytes) was analyzed by Spearman rank order correlation. Effect of teat shape, udder shape, and udder position were analyzed by one-way ANOVA.

## Results

### Effect of teat morphology

Table-1 describes the correlation between udder morphology with milk SCC, DLC and *in vitro* activity of milk leukocytes. There was a significant (p<0.01) positive correlation between milk SCC with mid teat diameter, teat base diameter and significant (p<0.05) negative correlation between milk SCC and the height of the teat from the ground. There was also a positive correlation between milk SCC and teat length and negative correlation between milk SCC and teat apex diameter though it was nonsignificant.

**Table-1:** Correlation between udder morphology and *in vitro* milk leukocyte activity in high yielding crossbred cows.

Parameters	SI	PI (N)	PI (M)	Teat length	Teat apex diameter	Mid teat diameter	Teat base diameter	Height from ground	Udder circumference	SCC	LYM	NEU	MAC
SI	1	−0.066	0.146	0.039	0.055	0.031	−0.035	−0.161	−0.056	0.103	0.093	−0.101	−0.012
PI (N)	−0.066	1	0.581**	−0.131	0.148	−0.072	−0.283**	0.102	−0.085	0.030	−0.196*	0.183	0.142
PI (M)	0.146	0.581**	1	0.02	0.332**	0.104	0.06	0.005	−0.055	0.055	−0.127	0.099	0.288**
Teat length	0.039	−0.131	0.02	1	−0.06	0.502**	0.607**	−0.502**	0.241*	0.178	0.107	−0.081	−0.029
Teat apex diameter	0.055	0.148	0.332**	−0.060	1	−0.001	−0.074	−0.061	−0.163	−0.122	−0.151	0.145	0.032
Mid teat diameter	0.031	−0.072	0.104	0.502**	−0.001	1	0.693**	−0.459**	0.108	0.258**	0.042	−0.012	−0.051
Teat base diameter	−0.035	−0.283**	0.06	0.607**	−0.074	0.693**	1	−0.368**	0.190	0.298**	0.096	−0.088	−0.030
Height from ground	−0.161	0.102	0.005	−0.502**	−0.061	−0.459**	−0.368**	1	−0.319**	−0.200*	−0.116	0.057	−0.006
Teat circumference	−0.056	−0.085	−0.055	0.241*	−0.163	0.108	0.19	−0.319**	1	−0.037	0.149	−0.082	0.317**
SCC	0.103	0.03	0.055	0.178	−0.122	0.258**	0.298**	−0.200*	−0.037	1	−0.227*	0.239*	−0.108
LYM	0.093	−0.196*	−0.127	0.107	−0.151	0.042	0.096	−0.116	0.149	−0.227*	1	−0.976**	0.172
NEU	−0.101	0.183	0.099	−0.081	0.145	−0.012	−0.088	0.057	−0.082	0.239*	−0.976**	1	−0.209*
MAC	−0.012	0.142	0.288**	−0.029	0.032	−0.051	−0.03	−0.006	0.317**	−0.108	0.172	−0.209*	1

* Significance at 5% level (p<0.05),

** Significance at 1% level (p<0.01). SI=Stimulation index, PI (N)=Phagocytic index of milk neutrophils, PI (M)=Phagocytic index of milk macrophages, LYM=Lymphocytes, NEU=Neutrophils, MAC=Macrophages, SCC=Somatic cell counts

There was no significant correlation between milk DLC and teat morphology. However, milk macrophage percentage was positively (p<0.01) correlated with udder circumference.

There was no significant correlation between SI of milk lymphocytes with teat morphology.

PI of milk neutrophil was found to be positively correlated with teat apex diameter and height of teat from ground. However, there was a significant (p<0.01) negative correlation between teat base diameter and PI of neutrophils.

PI of milk macrophages was nonsignificantly positively correlated with teat length, mid teat diameter, teat base diameter and height of teat from the ground and negatively correlated with udder circumference. However, there was a significant (p<0.01) positive correlation between *in vitro* PI of milk macrophages with teat apex diameter.

### Effect of teat shape

Effect of teat shape on milk SCC, DLC and *in vitro* milk leukocytic activity in high yielding crossbred cows have been presented in Table-2. Milk SCC did not differ significantly with different teat shape. However, it was nonsignificantly higher in pointed and cylindrical teats and lower in the case of round and flat teats. Milk DLC did not alter significantly with teat shape.

**Table-2:** Effect of teat shape on milk SCC, milk DLC and *in vitro* activity of milk leukocytes in high yielding crossbred cows.

Parameters	Teat shape			

	Pointed	Round	Flat	Cylindrical
Milk SCC (×10^5^ cells/ml)	2.19±0.24	1.62±0.38	1.51±0.35	2.15±0.11
Milk neutrophils (%)	57.04±5.30	52.51±8.50	48.47±7.80	46.18±2.50
Milk lymphocytes (%)	35.41±5.10	43.74±8.10	48.06±7.50	48.06±2.39
Milk macrophages (%)	5.76±0.5	3.74±0.9	4.73±0.8	5.01±0.2
Stimulation index lymphocytes	1.02±0.71	0.94±0.11	1.07±0.10	0.89±0.03
Phagocytic index neutrophils	0.81^a^±0.11	0.77^a^±0.18	0.29^b^±0.16	0.81^a^±0.05
Phagocytic index macrophages	0.94^a^±0.14	0.63^b^±0.22	1.10^b^±0.21	0.53^a^±0.06

Values are expressed as mean±SE. Values lack a common letter within a row differed significantly (p<0.05). SCC=Somatic cell counts, DLC: Differential leukocyte counts, SE: Standard error

Concanavalin A (Con A) induced milk lymphocyte blastogenic response was found to be unaltered with different teat shape. *In vitro* PI of milk neutrophils was found to be significantly (p<0.01) lower in flat teat. *In vitro* PI of milk macrophages was found to be significantly (p<0.01) lower in the round and flat teats compared to pointed and cylindrical teats.

### Effect of udder morphology

Effect of udder shape on milk SCC, DLC and *in vitro* milk leukocytic activity in high yielding crossbred cows have been presented in Table-3. Milk SCC was found to be significantly (p<0.01) lower in bowl-shaped udder compared to cup and round shaped udder. There was no significant difference in milk DLC with different udder shape except milk neutrophils where it was found to be significantly (p<0.05) lower in round shaped udder compared to cup and bowl-shaped udder. However, milk macrophage percentage was positively (p<0.01) correlated with udder circumference (Table-1).

**Table-3:** Effect of udder shape on milk SCC, milk DLC and *in vitro* activity of milk leukocytes in high yielding crossbred cows.

Parameters	Udder shape		

	Cup	Round	Bowl
Milk SCC (×10^5^ cells/ml)	2.36^x^±0.16	2.66^x^±0.25	1.83^y^±0.15
Milk neutrophils (%)	54.44^a^±2.86	41.20^b^±4.44	48.37^ab^±2.68
Milk lymphocytes (%)	40.54±2.82	51.74±4.37	46.06±2.64
Milk macrophages (%)	5.02±0.34	5.27±0.53	4.49±0.32
Stimulation index of milk lymphocytes	0.92±0.50	0.96±0.07	0.95±0.04
Phagocytic index of milk neutrophils	0.80±0.07	0.93±0.12	0.70±0.07
Phagocytic index of milk macrophages	0.68^ab^±0.09	0.92^a^±0.15	0.46^b^±0.09

Values are expressed as mean±SE. Values lack a common letter within a row differed significantly (p<0.05). SCC=Somatic cell counts, DLC: Differential leukocyte counts, SE: Standard error

Con A induced *in vitro* milk lymphocyte blastogenic responses were found to be unaltered with different udder shapes. *In vitro* PI of milk, neutrophil was found to be higher in round shaped udder compared to cup and bowl-shaped udder though the difference was nonsignificant. *In vitro* PI of milk macrophages was significantly (p<0.01) lower in bowl-shaped udder compared to round and cup-shaped udder.

### Effect of udder position

Effect of udder position on milk SCC, DLC and *in vitro* milk leukocytic activity in high yielding crossbred cows have been presented in Table-4. Milk SCC was significantly (p<0.01) higher in pendulous type udder compared to non pendulous udder type. There were no significant differences in milk DLC between different udder position, however, milk neutrophil were higher and milk lymphocytes were lower in pendulous type of udder compared to non-pendulous type of udder.

**Table-4:** Effect of udder position on milk SCC, milk DLC and *in vitro* activity of milk leukocytes in high yielding crossbred cows.

Parameters	Udder position	

	Non pendulous	Pendulous
Milk SCC (×10^5^ cells/ml)	1.32^a^±0.04	3.06^b^±0.05
Milk neutrophils (%)	45.75±2.20	52.51±2.90
Milk lymphocytes (%)	46.82±2.30	41.20±3.00
Milk macrophages (%)	5.37±0.26	5.06±0.36
Stimulation index lymphocytes	0.93±0.03	0.94±0.04
Phagocytic index neutrophils	0.84±0.05	0.70±0.07
Phagocytic index macrophages	0.73^a^±0.07	0.45^b^±0.09

Values are expressed as mean±SE. Values lack a common letter within a row differed significantly (p<0.05). SCC=Somatic cell counts, DLC: Differential leukocyte counts, SE: Standard error

There were no significant difference in ConA induced milk lymphocyte blastogenic response under different udder positions. *In vitro* PI of milk neutrophil did not differ significantly between different udder positions. However, it was lower in the pendulous type udder. The *in vitro* PI of milk macrophages was significantly p<0.01 lower in the pendulous type of udder compared to non pendulous type of udder.

## Discussion

The udder and teats are the first line of defense against intra-mammary infection, and the association between mastitis resistance and several udder type traits have been reviewed by many workers [6,8,11]. In this study, milk SCC was positively correlated with mid teat diameter, teat base diameter, and teat length which is in accordance with the earlier observations of Jørstad *et al*. [12] stated that teat canal diameter, teat injury and increased sphincter patency have a strong positive association with high SCC. Negative correlation between milk SCC and height of the teat from the ground in the present investigation was found to be similar with the findings of Saloniemi *et al*. [13] reported a short distance from the udder to the ground is associated with a predisposition to mastitis. Bhutto *et al*. [6] and Singh *et al*. [14] also reported decreasing teat end to floor distance, is a well-documented risk factor for mastitis.

To the best of our knowledge, this is the pioneer study correlating the udder morphology and *in vitro* activity of milk leukocytes. Here, we found PI of milk neutrophil and macrophages was positively correlated with teat apex diameter and height of teat from ground which could be explained by the fact that higher activity of milk neutrophils and macrophages in the case of higher teat to ground distance makes the mammary gland more resistance to intramammary infections. Con A induced milk lymphocyte blastogenic response could not be compared as no literature available in this regard.

The probability of mastitis occurring varies considerably between different teat shapes, sizes, teat placement, and the morphology of the teat tip [15]. In any case, there is no consensus in the literature about the influence of teat morphology on mastitis occurrence [14,16]. In the present investigation, phagocytic activity of milk neutrophils was negatively correlated with teat length, whereas Con A induced *in vitro* milk lymphocyte blastogenic response and PI of milk macrophages were negatively correlated. The earlier reports on the relationship between teat length and mastitis were also contradictory. Some authors suggested longer teats were positively correlated with mastitis incidence [17], whereas Slettbakk *et al*. [18] could not find an association between teat length and the risk of mastitis. In this study, a positive correlation found between milk SCC and teat length as the previous observation of Rogers *et al*. [11] reported positive correlations (0.16-0.20) between these two. However, Lund *et al*. [17] stated a very low genetic correlation (0.01) between teat length and SCC in contrast to others. These different findings could partly be explained by the interaction between liner and teat during milking. Both too short and too long teats were associated with an increased infection rate [19]. During this study, a significant positive correlation between teat diameter and milk SCC were found. Similarly, other workers also found an association teat diameter with the occurrence of mastitis [18].

These results suggest that the chances of mastitis are higher if the teat length is shorter and if the teat diameter is greater. There was a significant alteration in the *in vitro* activity of milk leukocytes under different teat shape. *In vitro* phagocytic activity of milk neutrophils and macrophages were lower in flat type teat compared to round and cylindrical teats. These findings were in accordance with the reports of Slettbakk *et al*. [18] and Bhutto *et al*. [6] reported inverted and less pointed teat as important risk factors for mastitis. Cows with inverted or pointed teat ends had higher SCC than cows with normal teat-end shapes [11]. However, Bakken [20] have also failed to find a relationship between mastitis susceptibility and teat-end shape.

The udder and teats are the first line of defense against intramammary infection. Udder morphology is very heritable and could serve as a marker trait for selection to reduce mastitis in dairy cattle [21,22]. Udder morphology has a significant influence on incidence of mastitis [7] as cows with less desirably shaped udders and more udder depth are more susceptible to lesions and contamination by mastitis-causing pathogens which increase the risk of mastitis. Here in this study, we found significantly (p<0.01) higher milk SCC and lower (p<0.01) phagocytic activity of milk macrophages in pendulous type of udder which was in accordance with the earlier observations of Slettbakk *et al*. [18] stated that high and strong udder attachment result in less clinical mastitis and lower SCC.

In this present investigation, we found higher activity of milk leukocytes and lower milk SCC in round shaped udder as reported by Saloniemi *et al*. [13] indicated that cows with dish-shaped or well-attached rounded udders have less mastitis than cows with a pendulous shaped udder. However, in contradiction, Hussain *et al*. [23] reported higher prevalence of mastitis in cattle having pendulous, round and bowl-shaped udder as long and pendulous udder gets injuries and helps the pathogens to grow.

## Conclusions

The study identified possible udder risk factors for incidence of mastitis such as teat shape and size, teat to floor distance, udder shape and size and found that these factors reduced the *in vitro* activity of milk leukocytes hence facilitates the incidence intramammary infections.

## Authors’ Contributions

JM planned the study. TS recorded the data. PRG and DB provided technical support and helped in data analysis. PKD analyzed the data. JM drafted and revised the manuscript. All authors read and approved the final manuscript.
